# Survey of Airborne Microorganisms in an Arcade-Type Traditional Market in Anseong, South Korea

**DOI:** 10.3390/ijerph19116667

**Published:** 2022-05-30

**Authors:** Chan-Geun Song, Jang-Hyun Park, Pum-Mook Lee, Myeong-Gyu Jung

**Affiliations:** 1 Korea Institute of Future Convergence Technology, Hankyong National University, 327 Jangang-ro, Anseong 17579, Korea; cgsong@hknu.ac.kr (C.-G.S.); parkjh@hknu.ac.kr (J.-H.P.); pm.lee@hknu.ac.kr (P.-M.L.); 2 Department of Industry-Academic Cooperation, Hankyong National University, Anseong 17579, Korea

**Keywords:** airborne, microorganism, arcade-type market, pathogenic bacteria, fungi

## Abstract

We aimed to analyze airborne microorganisms and assess air quality, temperature, and relative humidity at “J” Market, an arcade-type traditional market in Anseong (South Korea). Measurements were taken 16 times, twice per quarter (January, April, July, and October), at both the entrance and intersection of the market in 2020. The concentrations of airborne bacteria and fungi at the entrance and intersection were highest in October and lowest in April; however, they were below the recommended indoor levels (airborne bacteria: <800 CFU/m^3^, airborne fungi: <500 CFU/m^3^) in January (second measurement) and April (first and second measurements). The concentrations of microbes during the first measurement in January and both measurements in July and October exceeded the allowed limits. The concentration of microorganisms exceeded the acceptable levels at relative humidity ≥60%. At all time points, except during the eighth survey, when the microorganisms were too numerous to count, microbial concentrations were higher at the intersection than at the entrance. It was confirmed that the microorganisms detected in this experiment were 26 species of bacteria and 21 species of fungi. Three of the four species of bacteria and fungi detected in more than 50% of the 16 experimental results were pathogenic. Our findings suggest that air purification systems must be installed in the market to improve sanitary conditions.

## 1. Introduction

Traditional markets, originating from the term “conventional markets,” play a central role in the distribution of goods, not only as places for trading goods but also as comprehensive social, economic, and cultural spaces [1]. Such markets provide a socioeconomic base for local residents and revitalize the local economy [2]. However, problems such as the entry of large corporations and foreign companies into the distribution market, dilapidated buildings, and lack of amenities prevent these markets from keeping up with consumption patterns [3]. In order to revitalize distressed traditional markets in Korea, various revitalization projects have been implemented through agreements between the Korean Government and local municipalities over the past 10 years, starting with the enactment of the “Special Act on the Nurturing of Traditional Markets” in 2004 [4]. Consequently, economic recovery is being achieved with improvement in the physical environment, along with the use of tourism resources and tangible/intangible cultural content with local characteristics. In particular, a budget of over 1.5 trillion won has been allocated to change the structure of traditional markets to arcade-type markets to mitigate the physical effects that the weather has on traditional markets. However, arcade-type traditional markets have a semi-enclosed structure that makes them vulnerable to the effects of circulating air in regular outdoor environments. Depending on the level of ventilation in a traditional market with a high proportion of food ingredients and products, air stagnation and the presence of airborne microorganisms have negative health effects [5,6]. Despite various efforts to manage air quality in public use facilities where large groups of people gather, several management blind spots threaten the health of occupants.

Airborne microorganisms such as bacteria and fungi account for a high percentage of indoor air pollutants, along with fine dust [7]. Bacteria are self-reproducing biological pollutants that cause allergies and respiratory diseases and are usually found in food waste or dump yards. Fungi can exacerbate environmental diseases such as asthma, as well as other conditions such as atopic dermatitis and allergic rhinitis. The Korean Government has been managing airborne microorganisms that threaten indoor air quality through the enactment of the Indoor Air Quality Control in Public Use Facilities Act (Act No. 5224, 1996) and the Enforcement Decree of the Act (Presidential Decree No. 15,584, 1997). However, the management standards for semi-enclosed structures such as arcade-type traditional markets are ambiguous. Moreover, uncleanliness has been reported to be the main factor for the decrease in the number of users of public facilities such as arcade-type traditional markets [8]. Therefore, airborne microorganisms, which could have negative effects on public health, need to be managed in such spaces. Among studies on indoor air quality, basic research on the direct/indirect health effects of airborne microorganisms has been limited to hospitals and other indoor living spaces [9,10,11,12].

In the present study, we investigated the concentrations of airborne microorganisms in an arcade-type traditional market because it could be a blind spot in air quality management. The findings of this study could be used as basic data for policy development and the establishment of sanitary systems for traditional markets.

## 2. Materials and Methods

### 2.1. Study Site

The study was conducted at the arcade-type traditional market “J” Market in Anseong (Gyeonggi-do, Korea) in 2020. It is a semi-closed space that can be classified as a blind spot for air quality management owing to air stagnation. The market has four entrances where the air curtains are installed to reduce the inflow of outside air. The average monthly foot traffic is 38,700 persons, size of the market is 3086 m^2^, and total number of stores is 113. The market is cross-shaped with a horizontal length of 120 m and vertical length of 150 m. 

Airborne microorganisms in the market were analyzed 16 times: twice per quarter (January, April, July, and October) at the entrance and intersection of the market. The temperature and relative humidity at the entrance and intersection of the market were also recorded.

### 2.2. Air Sampling and Microbial Culture

An apparatus for collecting air samples must operate based on the impactor method [13]. Additionally, the flow rate of incoming air must be constant and measurable. In the present study, an MAS-100 Eco^®^ microbial air sampler (Merck, Darmstadt, Germany) was used for air sampling at a flow rate of 100 L/min (deviation: ±4%). The standard sample volume was adjusted to 50, 100, 250, 500, and 1000 L. The air sampler was made of anodized aluminum, and the air sampling filter had 400 holes. The size of each hole was φ110 mm × 180 mm. The air impact velocity was <20 m/s.

With respect to the air sampling method, air was sucked into the sampler at a constant flow rate through the sampling head plate, after which the sucked air was collected onto a microbial medium. The air sampler was placed on a tripod (height, 131 cm). Nutrient agar (NA) (KisanBio, Seoul, Korea, MB-N1036) and Sabouraud dextrose agar with chloramphenicol (SDAC) (Bandio, Pocheon-si, Korea, MO1112) were mounted on the tripod to collect the samples (collection concentration: 1000 L). Solid NA was used as the growth medium for bacteria as it supported the growth of a broad range of microorganisms, whereas SDAC was used for fungi. Chloramphenicol was added to the fungal growth medium to inhibit the growth of bacteria. The components of NA and SDAC are shown in Table 1.

Before the analysis of bacteria or fungi, the growth medium used was separated after sample collection and sealed to prevent external contamination. The bacterial colonies were counted after incubating the samples at 35 ± 1 °C for 24 h. In contrast, the fungal colonies were counted after incubating the samples at 25 ± 1 °C for 5 days. Proliferation was monitored, and the colonies were counted every 24 h to avoid difficulty in counting the fungal colonies as a result of fungal proliferation during incubation. The number of bacterial and fungal colonies was adjusted using a positive hole conversion table (Feller, 1950) to calculate the final microbial concentrations. Sample flow rate was calculated based on the average flow rate during sampling (Equation (1)) and the total volume of air sampled (Equation (2)) [14]. The concentrations of bacteria and fungi (colony forming unit [CFU]/m^3^) were calculated using Equation (3). The equations used are shown below.

Qave = (Q1 + Q2)/2(1)

where Qave is the average flow rate during sampling (L/min), Q1 is the flow rate at the start of sampling (L/min), and Q2 is the flow rate at the end of sampling (L/min).

V = (Qave × T)/10^3^ (2)

where V is the total volume of sampled air (m^3^) and T is the sampling time (min).

C = CFU/V(3)

where C is the total concentration of airborne microorganisms (CFU/m^3^) and CFU is the adjusted number of colonies.

### 2.3. Microbiological Identification and Analysis

#### 2.3.1. Pure Culture Isolation

**Figure 1:**
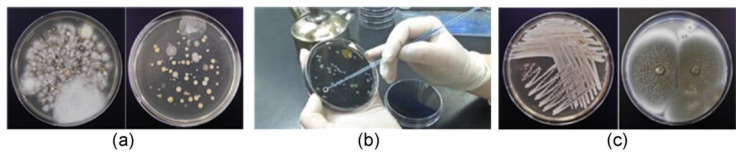
Procedure for pure culture isolation: (**a**) culturing of bacterial and fungal spores; (**b**) subculturing; (**c**) confirmation of pure culture isolation status.

After collecting the air samples, pure culture isolation was performed by subculturing bacterial and fungal spores present as cultured dominant species. The pure cultured bacterial and fungal spores were identified using molecular biological analysis. The steps for pure culture isolation are depicted in Figure 1. The isolation of pure culture was performed on an aseptic workbench to prevent external contamination. Sterilization of the workbench was achieved with 70% ethanol. A single colony or spore collected from the cultured bacteria or fungi using a loop was used to inoculate fresh media (NA and SDAC) for the first subculture. After inoculation, subculturing was carried out in an incubator at 35 ± 1 °C for 24 h for bacteria and at 25 ± 1 °C for 5−7 days for fungi. To check the status of pure culture isolation, we visually observed whether two or more types of bacteria and fungi were generated. Two or more colonies or spores showed different colors or formed a septum. A second subculture was performed when two or more colonies or spores were observed.

#### 2.3.2. Molecular Analysis

**Figure 2:**
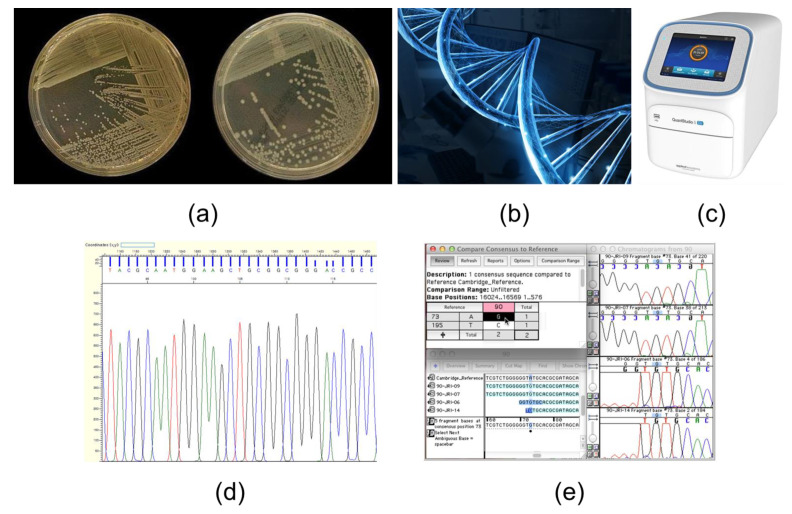
Procedure for the molecular analysis: (**a**) Preparation of pure isolates of bacteria and fungi; (**b**) gDNA extraction; (**c**) PCR equipment; (**d**) Sequencing analysis; (**e**) Sequence alignment.

Ribosomal RNA genes of pure isolates of bacteria and fungi were amplified for gene sequencing, and identification was performed using the National Center for Biotechnology Information (NCBI) rRNA database. Bacterial strains were identified via sequencing analysis using inter-primers 785F and 907R (Macrogen, Seoul, Korea) after 16S rRNA gene polymerase chain reaction (PCR) using 27F and 1492R primers (Macrogen). Fungi were identified via sequencing analysis after PCR using the internal transcribed spacer region. Figure 2 shows a summary of the procedure used in the experiment.

## 3. Results

The objective of the present study was to analyze the concentrations of airborne microorganisms in an arcade-type traditional market. Therefore, the concentration and type of airborne microorganisms in the air at the market were determined.

### 3.1. Concentration and Type of Airborne Microorganisms

Airborne microorganisms in the market were analyzed 16 times (twice per quarter at two points) in 2020. The concentrations of bacteria and fungi as well as the temperature and relative humidity at the entrance and intersection of the market are shown in Table 2.

#### 3.1.1. Concentrations of Airborne Microorganisms

The minimum and maximum concentrations of airborne bacteria at the entrance of the market were 6.30 × 10^2^ CFU/m^3^ and too numerous to count (TNTC), respectively, whereas those of airborne fungi were 4.50 × 10^2^ CFU/m^3^ and 7.00 × 10^2^ CFU/m^3^, respectively. The mean concentration of airborne bacteria at the entrance was the highest in October (9.30 × 10^2^ CFU/m^3^ and TNTC for the first and second measurements, respectively), followed by July (8.20 × 10^2^ CFU/m^3^), January (8.00 × 10^2^ CFU/m^3^), and April (6.40 × 10^2^ CFU/m^3^). Similarly, the mean concentration of airborne fungi was the highest in October (6.95 × 10^2^ CFU/m^3^), followed by July (6.65 × 10^2^ CFU/m^3^), January (5.40 × 10^2^ CFU/m^3^), and April (4.55 × 10^2^ CFU/m^3^).

The minimum and maximum concentrations of airborne bacteria at the intersection of the market were 6.90 × 10^2^ CFU/m^3^ and TNTC, respectively, whereas those of airborne fungi were 4.90 × 10^2^ CFU/m^3^ and 8.70 × 10^2^ CFU/m^3^, respectively. The quarterly concentrations of airborne bacteria and fungi at the intersection were similar to the results obtained at the entrance. The concentration of airborne bacteria was the highest in October (TNTC), followed by July (9.05 × 10^2^ CFU/m^3^), January (8.65 × 10^2^ CFU/m^3^), and April (7.30 × 10^2^ CFU/m^3^). The concentration of airborne fungi was also the highest in October (8.55 × 10^2^ CFU/m^3^), followed by July (7.20 × 10^2^ CFU/m^3^), January (5.90 × 10^2^ CFU/m^3^), and April (4.90 × 10^2^ CFU/m^3^).

The minimum and maximum temperature readings at the entrance of the market were −2.1 °C and 27.0 °C, respectively, whereas the minimum and maximum relative humidity readings were 48.9% and 80.8%, respectively. The average temperature at the intersection (15.3 °C) was higher by 1.2 °C than that at the entrance (14.1 °C), whereas the average relative humidity at the intersection (62.1%) was higher by 0.1% than that at the entrance (62.0%).

**Figure 3:**
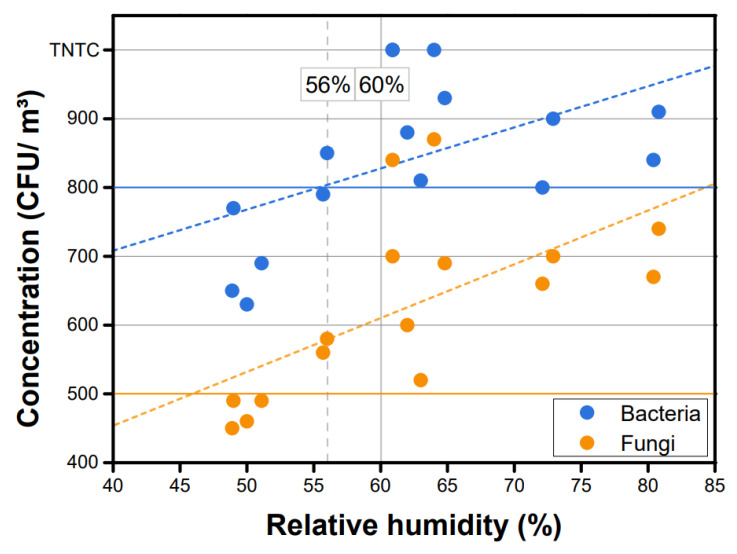
Concentrations of airborne microorganisms with respect to relative humidity.

As shown in Figure 3, the relative humidity obtained at both entrance and intersection was the highest in July (first reading, 72.1%; second reading, 80.4%), followed by October, January, and April, whereas the lowest relative humidity was recorded in April (first reading, 48.9%; second reading, 50.0%). The average concentration of airborne bacteria could not be calculated because the bacteria were TNTC in October. The average concentration of fungi was 5.88 × 10^2^ CFU/m^3^ at the entrance and 6.63 × 10^2^ CFU/m^3^ at the intersection.

#### 3.1.2. Identification of Airborne Microorganisms

A total of 47 species of airborne microorganisms were detected during the 16 experiments at the entrance and intersection of the market. The frequency of detection of the microorganisms ranged between 1 and 16. As shown in Table 3, 26 species of airborne bacteria were detected in the study. *Bacillus subtilis*, *Bacillus licheniformis*, *Bacillus megaterium*, and *Staphylococcus xylosus* were detected in all 16 experiments. Nine different bacterial species were detected only once, another nine were detected twice, one species was detected in three experiments, and three species were detected five times.

As shown in Table 4, 21 species of airborne fungi were detected in the study. The results show that *Aspergillus niger* and *Fusarium sporotrichioides* were detected in all 16 experiments. *Penicillium variabile* and *Rhizomucor pusillus* were detected eight times. One fungal species was detected four times, five species were detected three times, four were detected two times, and seven were detected just once.

## 4. Discussion

Most traditional markets in Korea are designed as semi-enclosed arcade-type structures. As a result, they are usually poorly ventilated unless special equipment has been installed to maintain ventilation. There is natural ventilation in such markets, unlike in indoor environments; however, there could be areas with poor air circulation depending on the structural characteristics of the facility. Areas with poor ventilation or humid environments may have negative health effects because of high concentrations of airborne microorganisms such as bacteria and fungi in such areas.

According to a press release from the Korean Ministry of Environment, the total concentration of airborne bacteria in indoor public use facilities should not exceed 800 CFU/m^3^, whereas the recommended limit for airborne fungi by the World Health Organization is 500 CFU/m^3^. The present study site was an arcade-type traditional market, and such a semi-enclosed quasi-indoor space does not have any standards with respect to air quality. Accordingly, standards for indoor public use facilities were used for comparison in this study. Our results showed that the levels of microorganisms detected exceeded the standard limits, except for the levels determined in April (first and second measurements) and January (second measurement). The highest and lowest levels were obtained in October and April, respectively. These findings are similar to the results from a 2011 survey on airborne microorganisms in residential homes reported by the Ministry of Environment [15]. These results are considered to be related to the proliferation of airborne microorganisms in damp environments during the summer monsoon.

It is recommended that the relative humidity should be maintained at ≤60% for pleasant air quality [15]. The studies that detected indoor airborne microorganisms concentration have also confirmed that the concentration of indoor airborne microorganisms is high depending on humidity [9,16]. The findings of the present study showed a relative humidity value of 60%, exceeding the standard level required for the concentration of indoor airborne microorganisms to be low. As shown in Figure 3, the concentrations of both bacteria and fungi were below their respective limits at a relative humidity below 56% but above their standard limits at relative humidity above 56%.

The arcade-type traditional market is a semi-enclosed space; therefore, it represents an environment with air quality similar to that of indoor air. Moreover, meat and other food products are mostly sold at traditional markets; thus, the proliferation of airborne bacteria and fungi is likely to occur during the humid summer and winter. This suggests that there is a need to control the humidity in such areas through the use of indoor ventilation and purification systems to prevent the environment from getting humid.

**Figure 4:**
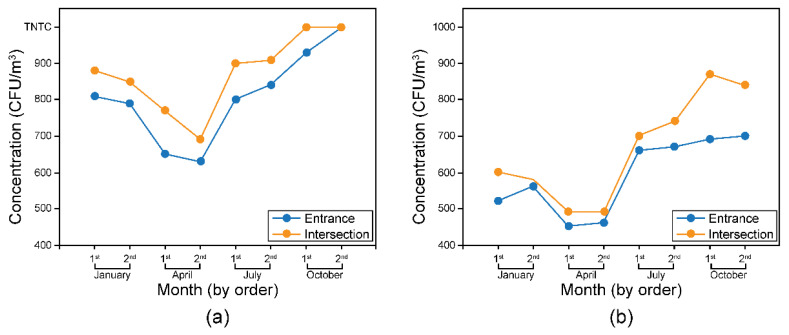
Concentrations of airborne microorganisms at the entrance and intersection of the market: (**a**) bacteria; (**b**) fungi.

As shown in Figure 4, the concentrations of bacteria and fungi were higher at the intersection than at the entrance of the market at all time points except in October (TNTC). “J” Market is a cross-shaped facility; thus, the intersection represents a point at which winds from all directions gather. We believe that the concentration of airborne microorganisms was higher at the intersection because there was a direct inflow of outdoor air to that area. Moreover, the intersection is the point with high air stagnation and heavy foot traffic.

Products are not usually sold in groups in traditional markets, unlike in supermarkets. This is because stores at which meat, snacks, and general merchandise are sold are often mixed together. Therefore, additional studies may be needed to compare the levels of airborne microorganisms according to store concentration.

A total of 26 airborne bacterial species were detected in the present study, with four species being detected in all 16 experiments. Of these four species, *B. subtilis* is non-pathogenic; however, it can cause eye or urinary tract infections in humans and is widely distributed in nature, as it can be found in the air, soil, and grass [17]. *Bacillus licheniformis* can cause infection in immunocompromised patients and has been identified as a cause of ventriculitis, ophthalmitis, and peritonitis. Additionally, it has been identified as a contaminant in dairy products [18]. *Bacillus megaterium* inhibits the growth of harmful bacteria, promotes the proliferation and growth of beneficial bacteria, maintains the dynamic balance of intestinal flora, protects intestinal health, and inhibits pathogens [19]. *Staphylococcus xylosus* is a gram-positive bacterium; some strains are pathogenic and resistant to various antibiotics, and they can cause female urinary tract infection and mouse skin lesions [20].

A total of 21 species of fungi were detected, with four species detected in at least eight experiments. Among the detected species, *A. niger* is a pathogen of onions, peanuts, and grapes. It can also cause otomycosis, which is characterized by ear pain, temporary hearing loss, and eardrum damage [21]. *Fusarium sporotrichioides* is a pathogen of grains such as wheat, barley, oats, corn, and rye. Although *F. sporotrichioides* primarily infects crops, consuming infected crops could have negative health effects due to mycotoxins [22]. *Penicillium variabile* is found in wheat, flour, corn, rice, and barley, as well as in indoor environments [23]. *Rhizomucor pusillus* causes zygomycosis in humans and diseases in the lungs of immunocompromised patients. It is reported that *R. pusillus* infections can even cause death. This fungus is also pathogenic in animals and can cause mucormycotic abortion [24].

There is usually heavy foot traffic at traditional markets. Additionally, various bacteria are present at such markets. As a result, there is a high likelihood of various types of airborne microorganisms being found in traditional markets. Although 26 species of airborne bacteria and 21 species of airborne fungi were detected in this study, airborne microorganisms detected at low levels and suspected to have been introduced by outside sources were excluded from the analysis. Table 5 shows the eight species of airborne microorganisms detected in more than eight experiments out of 16 measurements (≥50%).

Among the eight species of microorganisms, six were pathogenic, indicating that they could have negative effects on humans. Risk factors that could accelerate decay by directly affecting food and those responsible for respiratory and immunological disorders were also present. Pathogenic airborne microorganisms can cause infectious and allergic diseases and even death in severe cases [25]. Many pathogenic and non-pathogenic airborne microorganisms are present in arcade-type traditional markets, and they can threaten the health of merchants and visitors. Efforts are needed to improve air quality at traditional markets for the health of occupants, and awareness to maintain such markets clean must be created. Structural improvements such as the installation of air purification systems and ventilation systems, as well as regular testing of the effects of such systems, are necessary.

## 5. Conclusions

The concentrations of bacteria and fungi were the highest in October and the lowest in April at both entrance and intersection. The results indicate that the concentrations of microorganisms were higher during summer and winter than during dry spring. When the relative humidity was ≥55%, the concentrations of the microorganisms exceeded their standard limits; this finding corroborated the recommendation by the Ministry of Environment to maintain indoor relative humidity at ≤60%. Microorganism concentrations were mostly higher at the intersection than at the entrance of the market.

A total of 47 airborne microbial species were detected in the study: 26 bacterial species and 21 fungal species. Three species of bacteria and fungi, respectively, detected at eight or more time points were pathogenic, whereas one species each was non-pathogenic. The results show that the air quality at “J” Market in 2020, with respect to the concentrations of microorganisms, varied depending on the season and relative humidity, often exceeding the recommended standard limits.

Although this study does not detect the specific airborne microorganisms and does not have enough references to compare the data from similar spots to this study site, the findings of this study could be beneficial in implementing sanitation management strategies in similar types of traditional markets.

## Figures and Tables

**Table 1:** Components of the growth media.

Medium	Component	Amount
NA	Bacto peptone	5.00 g
	Beef extract	3.00 g
	Agar	15.00 g
	Distilled water	1000 mL
SDAC	Enzymatic digest of casein	5.00 g
	Enzymatic digest of animal tissue	5.00 g
	Dextrose	40.00 g
	Agar	15.00 g
	Chloramphenicol	0.05 g
	Distilled water	1000 mL

NA: nutrient agar; SDAC: Sabouraud dextrose agar with chloramphenicol.

**Table 2:** Concentrations of different airborne microorganisms at “J” Market.

Location	Category	January		April		July		October	
		1st (01/15)	2nd (01/30)	1st (04/14)	2nd (04/29)	1st (07/15)	2nd (07/30)	1st (10/15)	2nd (10/30)
Entrance	Temperature (°C)	−2.1	−1.7	13.4	16.2	24.3	26.1	19.4	17.1
	Relative humidity (%)	63.0	55.7	48.9	50.0	72.1	80.4	64.8	60.9
	Bacterial concentration (CFU/m^3^)	8.1 × 10^2^	7.9 × 10^2^	6.5 × 10^2^	6.3 × 10^2^	8.0 × 10^2^	8.4 × 10^2^	9.3 × 10^2^	TNTC
	Mean bacterial concentration [CFU/m^3^] (SD)	8.0 × 10^2^ (14.1)		6.4 × 10^2^ (14.1)		8.2 × 10^2^ (28.3)		-	
	Fungal concentration (CFU/m^3^)	5.2 × 10^2^	5.6 × 10^2^	4.5 × 10^2^	4.6 × 10^2^	6.6 × 10^2^	6.7 × 10^2^	6.9 × 10^2^	7.0 × 10^2^
	Mean fungal concentration [CFU/m^3^] (SD)	5.4 × 10^2^ (28.3)		4.55 × 10^2^ (7.1)		6.65 × 10^2^ (14.1)		6.95 × 10^2^ (7.1)	
Intersection	Temperature (°C)	−1.0	0.9	14.4	17.3	25.1	27.0	20.3	18.3
	Relative humidity (%)	62.1	56.0	49.0	51.1	72.9	80.8	64.0	60.9
	Bacterial concentration (CFU/m^3^)	8.8 × 10^2^	8.5 × 10^2^	7.7 × 10^2^	6.9 × 10^2^	9.0 × 10^2^	9.1 × 10^2^	TNTC	TNTC
	Mean bacterial concentration [CFU/m^3^] (SD)	8.65 × 10^2^ (21.2)		7.3 × 10^2^ (56.6)		9.05 × 10^2^ (7.1)		-	
	Fungal concentration (CFU/m^3^)	6.0 × 10^2^	5.8 × 10^2^	4.9 × 10^2^	4.9 × 10^2^	7.0 × 10^2^	7.4 × 10^2^	8.7 × 10^2^	8.4 × 10^2^
	Mean fungal concentration [CFU/m^3^] (SD)	5.9 × 10^2^ (14.1)		4.9 × 10^2^ (0.0)		7.2 × 10^2^ (28.3)		8.55 × 10^2^ (21.2)	

CFU, colony forming unit; SD, standard deviation; TNTC, too numerous to count.

**Table 3:** Detection frequency of airborne bacteria.

No.	Bacterial Species	Detection Frequency
1	*Bacillus subtilis*	16/16 (100%)
2	*Bacillus licheniformis*	16/16 (100%)
3	*Bacillus megaterium*	16/16 (100%)
4	*Staphylococcus xylosus*	16/16 (100%)
5	*Brevibacterium frigoritolerans*	5/16 (31%)
6	*Bacillus vallismortis*	5/16 (31%)
7	*Pantoea agglomerans*	5/16 (31%)
8	*Kocuria rosea*	3/16 (19%)
9	*Bacillus altitudinis*	2/16 (13%)
10	*Bacillus thuringiensis*	2/16 (13%)
11	*Exiguobacterium acetylicum*	2/16 (13%)
12	*Arthrobacter aurescens*	2/16 (13%)
13	*Bacillus velezensis*	2/16 (13%)
14	*Deinococcus soli*	2/16 (13%)
15	*Arthrobacter luteolus*	2/16 (13%)
16	*Psychrobacter faecalis*	2/16 (13%)
17	*Staphylococcus equorum*	2/16 (13%)
18	*Exiguobacterium indicum*	1/16 (6%)
19	*Acinetobacter schindleri*	1/16 (6%)
20	*Bacillus zhangzhouensis*	1/16 (6%)
21	*Bacillus cereus*	1/16 (6%)
22	*Bacillus flexus*	1/16 (6%)
23	*Bacillus muralis*	1/16 (6%)
24	*Bacillus wiedmannii*	1/16 (6%)
25	*Exiguobacterium sibiricum*	1/16 (6%)
26	*Kocuria rhizophila*	1/16 (6%)

**Table 4:** Detection frequency of airborne fungi.

No.	Fungal Species	Detection Frequency
1	*Aspergillus niger*	16/16 (100%)
2	*Fusarium sporotrichioides*	16/16 (100%)
3	*Penicillium variabile*	8/16 (50%)
4	*Rhizomucor pusillus*	8/16 (50%)
5	*Rhizopus microsporus*	4/16 (25%)
6	*Aspergillus fumigatus*	3/16 (19%)
7	*Penicillium citrinum*	3/16 (19%)
8	*Phoma* sp.	3/16 (19%)
9	*Alternaria alternata*	3/16 (19%)
10	*Penicillium* sp.	3/16 (19%)
11	*Fungal endophyte*	2/16 (13%)
12	*Irpex lacteus*	2/16 (13%)
13	*Aspergillus luchuensis*	2/16 (13%)
14	*Aspergillus oryzae*	2/16 (13%)
15	*Curvularia lycopersici*	1/16 (6%)
16	*Emericella dentata*	1/16 (6%)
17	*Aspergillus* sp.	1/16 (6%)
18	*Emericella* sp.	1/16 (6%)
19	*Penicillium janthinellum*	1/16 (6%)
20	*Trichoderma longibrachiatum*	1/16 (6%)
21	*Trichoderma* sp.	1/16 (6%)

**Table 5:** Airborne microbial species with detection frequencies of ≥50%.

Category	Pathogenicity	Microorganism	Pure Culture Isolation	Characteristics
Bacteria	Pathogenic	*Bacillus subtilis*	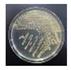	Partially pathogenic Causes eye and urinary tract infections
		*Bacillus licheniformis*	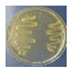	Pathogenic Causes an infection in immunocompromised patients
		*Staphylococcus xylosus*	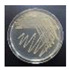	Partly pathogenic Causes urinary tract infection in females and skin lesions
	Non-pathogenic	*Bacillus megaterium*	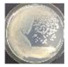	Inhibits the growth of pathogenic microorganisms
Fungi	Pathogenic	*Aspergillus niger*	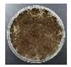	Pathogenic Causes infection in the ear canal
		*Fusarium sporotrichioides*	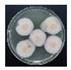	Pathogenic Fungal toxins have negative effects in humans
		*Rhizomucor pusillus*	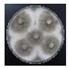	Pathogenic Causes lung disease
	Non-pathogenic	*Penicillium variabile*	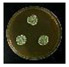	Exists in the indoor environment

## Data Availability

The data used and/or analyzed during the current study are available from the corresponding author on request.
